# Hyperthyroidism‐Induced Nondiabetic Ketoacidosis: A Rare Case Report

**DOI:** 10.1002/ccr3.9698

**Published:** 2025-02-19

**Authors:** Elias Battikh, Sherif Mostafa, Hadeel Alfar, Koutaibah Obaid, Ashraf I. Ahmed, Bisher Sawaf, Dabia Al‐Mohanadi

**Affiliations:** ^1^ Department of Internal Medicine Hamad Medical Corporation Doha Qatar; ^2^ The John H. Stroger Jr. Hospital of Cook County Chicago Illinois USA; ^3^ College of Medicine, QU Health Qatar University Doha Qatar

**Keywords:** hyperthyroid, nondiabetic ketoacidosis (NDKA), thyroid stimulating hormone, thyrotoxicosis

## Abstract

Hyperthyroidism, marked by excess thyroid hormones (T4 and T3), presents with symptoms like palpitations, heat intolerance, anxiety, and weight loss despite a normal or increased appetite. Diagnosis involves low serum TSH levels and elevated free T4 and/or T3. Rarely, hyperthyroidism can lead to nondiabetic ketoacidosis (NDKA). This report describes a 40‐year‐old woman with palpitations, chest discomfort, heat intolerance, insomnia, and significant weight loss. Laboratory tests showed severely suppressed TSH, elevated free T4 and T3, and high anion gap metabolic acidosis with elevated ketone levels. She was diagnosed with hyperthyroidism‐induced NDKA. Hyperthyroidism can promote lipolysis and ketogenesis, potentially leading to NDKA. Though uncommon, it is essential for clinicians to consider hyperthyroidism in NDKA cases when common causes are excluded. Our patient's symptoms significantly improved with propranolol and carbimazole treatment. This case emphasizes the importance of recognizing hyperthyroidism as a potential cause of NDKA. Appropriate diagnosis and treatment with antithyroid medications can lead to complete symptom resolution and normalization of biochemical markers.

**Summary**

- This case highlights the rare occurrence of nondiabetic ketoacidosis as a manifestation of undiagnosed thyrotoxicosis.
- In essence, hyperthyroidism can lead to severe metabolic complications, including ketoacidosis, which is typically seen in diabetic patients.
- It is crucial for clinicians to maintain a high index of suspicion for thyroid dysfunction in patients presenting with ketoacidosis without diabetes, as timely diagnosis and treatment of hyperthyroidism can lead to rapid resolution of symptoms and metabolic abnormalities.

**Abbreviations**
NDKA: nondiabetic ketoacidosis
T4/T3: thyroid hormones (Thyroxine)

## 1 Introduction

Hyperthyroidism is a hormonal imbalance characterized by an excess of thyroid hormones, including thyroxine (T4) and triiodothyronine (T3) [1]. Symptoms of hyperthyroidism encompass tremors, palpitations, heat intolerance, increased perspiration, anxiety, emotional fluctuations, weakness, and weight loss despite a normal or heightened appetite [1]. The diagnosis of primary hyperthyroidism is based on symptoms and biochemical evidence of hyperthyroid state, which includes low serum thyroid‐stimulating hormone levels and elevated free T4 and/or T3 concentrations [2]. Hyperthyroid state and excess thyroid hormones are associated with several complications that include but not limited to heart failure, systolic hypertension, atrial fibrillation, osteoporosis, weight loss due to an accelerated metabolic rate and a potentially life‐threatening condition called thyrotoxicosis [3]. Moreover, several case reports suggested that hyperthyroidism may rarely lead to nondiabetic ketoacidosis (NDKA). This case report details an instance of undiagnosed thyrotoxicosis initially manifesting with typical hyperthyroidism symptoms of palpitations, heat intolerance, and weight loss. Furthermore, work up showed NDKA state. This case represents one of few cases of hyperthyroidism‐induced NDKA reported worldwide, aiming to increase awareness of this uncommon condition and delineate our proposed management approach.

## 2 Case Presentation

### 2.1 Clinical Presentation

A 40‐year‐old lady with no past medical history presented to the emergency department with palpitations and chest discomfort. She reported ongoing symptoms for 1 week prior to admission. The patient's symptoms had started gradually and were increasing in intensity, which prompted her to seek medical attention. She also reported long standing history of heat intolerance, insomnia, and sleep disturbances. Further questioning revealed that the patient had experienced an unintentional 5‐kg weight loss in the month preceding her admission. Furthermore, she reported history of menstrual irregularity for the past 2 months. However, she denied having diarrhea, excessive sweating, hair loss, or tremors.

The patient denied any other past medical history or any personal history of similar episodes. Additionally, the patient did not report any prior hospital admissions. Moreover, she denied family history of thyroid disease or tumors. As for her social history, she works as a housemaid, does not drink alcohol, smoke, or consume any recreational drinks or drugs.

On presentation, she was afebrile, conscious, alert, oriented, maintaining saturation of 99% on room air with a heart rate of 129 bpm and blood pressure of 138/88 mmHg, patient weighted 61 kg and BMI of 24.1. Clinical examination revealed regular peripheral pulse with bilateral fine hand tremors. Further physical examination revealed a nontender, diffusely enlarged thyroid gland without any palpable nodules. Eye examination revealed periorbital edema, mild lid lag, retraction, and exophthalmos bilaterally. The Cardiac exam revealed normal S1 and S2 with no added murmurs. The neurological exam was not significant for any focal neurological deficit. Abdominal examination revealed a soft and lax abdomen, with no tenderness, overlying skin changes, organomegaly, or renal artery bruits. No skin lesion was identified on examination, and mucous membranes were intact with no discoloration or lesions. The musculoskeletal exam of the major joints (neck, shoulder, elbow, hip, knee, and ankle) did not reveal any proximal muscle weakness, tenderness or limitation in the range of motion in the joints. Furthermore, she did not have lower limb edema or discoloration.

### 2.2 Investigation and Treatment

Laboratory tests (Table 1) revealed severely suppressed TSH of < 0.01 mIU/L with high free T4 of 72 pmol/L and high free T3 of 21.5 pmol/L. TSH receptor antibody came back positive with a value of 19.7 IU/L. Moreover, venous blood gas analysis (Table 2) showed a picture of high anion gap metabolic acidosis with pH of 7.24, bicarbonate low at 9.8, pCO_2_ of 23 mmHg, sodium of 141 mmol/L and chloride of 113 mmol/L. Calculated anion gap was around 18. Work up for high anion gap metabolic acidosis showed elevated urine ketones, elevated serum beta‐hydroxybutyrate of 4.63 mmol/L, normal lactic acid, normal random blood sugar, and normal HbA1C levels. Ethanol, urea, and salicylate levels were also within normal limits.

**TABLE 1:** Shows complete metabolic count, blood chemistry, and toxicology screening done on admission.

Blood test	Value w/units	Normal range
Salicylate levels	< 0.3 mg/dL	< 0.3 mg/dL
Beta hydroxy butyrate	4.63 mmol/L	0.03–0.3 mmol/L
HbA1C %	4.2%	< 6.5%
TSH receptor antibody (anti‐TSHR)	19.7 IU/L	< 1.75 IU/L
Ethanol	< 2 mmol/L	< 2 mmol/L
WBC	7.7 × 10^3^/μL	4.0–10.0 × 10^3^/μL
RBC	5.4 × 10^6^/μL	3.8–4.8 × 10^6^/μL
Hgb	14.4 g/dL	12.0–15.0 g/dL
Hct	43.5%	36.0%–46.0%
MCV	81.0 fL	83.0–101.0 fL
MCH	26.8 pg	27.0–32.0 pg
Platelet	254 × 10^3^/μL	150–410 × 10^3^/μL
Urea	4.2 mmol/L	2.5–7.5 mmol/L
Creatinine	54 μmol/L	40–80 μmol/L
Calcium	2.31 mmol/L	2.20–2.60 mmol/L
Adjusted calcium	2.41 mmol/L	2.20–2.60 mmol/L
Bilirubin T	12 μmol/L	0–21 μmol/L
Total protein	82 g/L	60–80 g/L
Albumin lvl	35 g/L	35–50 g/L
Alk phos	104 U/L	35–104 U/L
ALT	21 U/L	0–33 U/L
AST	36 U/L	10–40 U/L
TSH	< 0.01 mIU/L	0.30–4.20 mIU/L
Free T3	21.5 pmol/L	3.7–6.4 pmol/L
Free T4	72.0 pmol/L	11.0–23.3 pmol/L

**TABLE 2:** Shows initial venous blood gas analysis.

Blood test	Value w/units	Normal range
pH ven‐POC	7.244	7.320–7.420
PCO2 ven‐POC	23 mmHg	41–51 mmHg
PO2 ven‐POC	60 mmHg	25–40 mmHg
Na ven‐POC	141 mmol/L	135–145 mmol/L
K ven‐POC	4.2 mmol/L	3.5–5.0 mmol/L
Cl ven‐POC	113 mmol/L	96–110 mmol/L
BG glu ven‐POC	3.4 mmol/L	3.3–5.5 mmol/L
BG lac ven‐POC	1.30 mmol/L	0.50–2.20 mmol/L
tHb ven‐POC	15.7 g/dL	12.0–16.0 g/dL
HCO_3_ ven‐POC	9.8 mmol/L	23.0–29.0 mmol/L

ECG revealed sinus tachycardia with no acute ischemic changes or other conduction abnormalities. Echocardiography revealed normal ejection fraction, normal valvular anatomy and no evidence of regional wall motion abnormalities. CT head was ordered as well and showed chronic microangiopathic changes with no recent acute ischemic infarction. Ultrasound of the thyroid revealed diffusely decreased echogenicity with a slightly heterogeneous texture and increased vascularity throughout the thyroid parenchyma. No focal lesions or nodules were identified. The clinical and laboratory findings were consistent with a diagnosis of Graves' disease.

The patient was initially administered a 500 mL bolus of normal saline for fluid resuscitation. Following this, she was placed on an infusion of 1000 mL of NS at a rate of 250 mL per hour. To address the metabolic acidosis, 150 mEq of sodium bicarbonate (NaHCO₃) was mixed with 850 mL of 5% dextrose water (D5W) and administered at a rate of 100 mL per hour. The total fluid administered during resuscitation amounted to 2.5 L. She was also given 200 mg of intravenous hydrocortisone, 80 mg of sustained release propranolol and 20 mg of carbimazole for treatment of her hyperthyroidism.

### 2.3 Outcome and Follow‐Up

She was subsequently hospitalized for observation and further management. During hospitalization she was kept on propranolol 20 mg and carbimazole 20 mg twice daily. Her symptoms improved with improvement in pH and bicarbonate values. On day 2 of hospitalization, the anion gap closed, and her pH returned back to normal value. The patient was discharged after 4 days of hospitalization in stable conditions, continuing on propranolol and carbimazole treatment at home. She was also given a referral to the endocrine clinic for further follow up but she was lost to follow up.

## 3 Discussion

Hyperthyroid state is characterized by several, nonspecific and systemic signs and symptoms [4]. Hyperthyroidism, a condition characterized by excessive production and secretion of thyroid hormones, represents a group of disorders involving elevated thyroid hormone action at the tissue level due to increased thyroid hormone concentrations in the serum. In the USA, the prevalence of hyperthyroidism is 1.2%, with overt hyperthyroidism accounting for 0.5% and subclinical hyperthyroidism for 0.7% [5]. Graves' disease is the most common cause of hyperthyroidism in iodine‐replete areas, predominantly affecting women aged 30 to 60 years. Relatively, ketoacidosis is a metabolic state characterized by abnormally high serum and urine ketone body concentrations. It is broadly categorized into diabetic ketoacidosis and NDKA [6]. NDKA occurs in the absence of diabetes and is associated with favorable outcomes and almost complete recovery in all cases. Causes of NDKA include starvation, alcoholism, pregnancy, lactation, and a low‐carbohydrate diet [7]. Thyrotoxicosis is one of the rarest causes of NDKA [8].

In our patient, NDKA was presented with an unknown cause. Initially, the focus was to find the cause of NDKA and treat the patient accordingly. However, initial evaluation of ketoacidosis was unremarkable. Laboratory values of random blood glucose, HbA1C, lactic acid, urea, ethanol, and salicylic acid were all within normal limits. Nevertheless, our patient had clinic signs and symptoms of hyperthyroidism with biochemical evidence confirming hyperthyroid state. After initiation of propranolol and carbimazole, the patient experienced significant symptom improvement. In alignment with the literature, we identified only two adult cases and one pediatric case of hyperthyroidism with NDKA, indicating that elevated TH levels are a primary trigger for ketoacidosis [8]. Notably, all three cases, including ours, showed a remarkable response to antithyroid medication and hydration with complete resolution of symptoms and normalization of serum pH.

The underlying mechanism of the observed clinical picture in our patient remains poorly understood. Nevertheless, it may be attributed because of the influence of thyroid hormones on lipolysis through various mechanisms, including increased beta‐2 adrenergic receptors and lipolytic response in adipocytes [9]. Furthermore, Elevated norepinephrine in subcutaneous adipose tissue of thyrotoxicosis patients may stimulate lipolysis by enhancing local norepinephrine release. Additionally, thyroid hormones upregulate key enzymes in the carnitine shuttle system, such as carnitine palmitoyl transferase (CPT), which facilitates the transport of long‐chain fatty acids into liver cell mitochondria for beta‐oxidation [10] This enhanced beta‐oxidation leads to increased production of ketone bodies, thereby contributing to the development of ketoacidosis. Moreover, thyroid hormones are known to exert nongenomic effects on mitochondria, increasing oxygen consumption and ATP production, which further accelerates fatty acid breakdown [10]. These combined effects of elevated thyroid hormone levels stimulate an excess release of free fatty acids into circulation, which are subsequently oxidized, promoting ketogenesis. Relatively, there is growing evidence that thyroid hormones play a role in stimulating hepatic gluconeogenesis, which could exacerbate the metabolic shift toward fat oxidation, particularly in a hyperthyroid state. In thyrotoxicosis, increased glucose production combined with insulin resistance enhances the body's reliance on lipolysis for energy, resulting in further ketone body production [11].

These mechanisms collectively support the role of thyroid hormones in promoting lipolysis and fatty acid release from adipocytes, leading to ketoacidosis as seen in our case and previously documented cases. Herin, we highlight the potential role of thyrotoxicosis in inducing severe ketoacidosis, emphasizing the importance of early diagnosis and intervention in hyperthyroid patients presenting with metabolic acidosis.

## 4 Conclusion

In conclusion, hyperthyroidism is a condition characterized by excessive thyroid hormone production, leading to a range of systemic symptoms. Ketoacidosis is a metabolic state with abnormally high ketone levels, categorized as diabetic or nondiabetic (NDKA). NDKA can occur without diabetes, often because of conditions like starvation or low‐carb diets. Thyrotoxicosis is a rare cause of NDKA, as thyroid hormones can promote lipolysis and ketogenesis. In this reported case, the patient presented with NDKA of unknown origin, but further evaluation revealed hyperthyroidism as the underlying cause. Treatment with antithyroid medications led to symptom resolution, highlighting the importance of considering thyroid disorders as a potential trigger for NDKA.

## Author Contributions

**Elias Battikh:** conceptualization, investigation, methodology, validation, visualization, writing – original draft, writing – review and editing. **Sherif Mostafa:** conceptualization, investigation, methodology, validation, visualization, writing – original draft, writing – review and editing. **Hadeel Alfar:** conceptualization, investigation, methodology, validation, visualization, writing – original draft, writing – review and editing. **Koutaibah Obaid:** conceptualization, investigation, methodology, validation, visualization, writing – original draft, writing – review and editing. **Ashraf I. Ahmed:** conceptualization, validation, visualization, writing – original draft, writing – review and editing. **Bisher Sawaf:** conceptualization, validation, visualization, writing – original draft, writing – review and editing. **Dabia Al‐Mohanadi:** conceptualization, data curation, methodology, project administration, supervision, validation, visualization, writing – original draft, writing – review and editing.

## Ethics Statement

The article was approved by the Institution Review Board at Hamad Medical Corporation.

## Consent

A written informed consent was obtained from the patient for the publication of all images, clinical data, and other data included in the manuscript. All identifying information has been removed.

## Conflicts of Interest

The authors declare no conflicts of interest.

## Data Availability

The data that support the findings of this study are available from the corresponding author upon reasonable request.
